# Evidence of a Role for One-Carbon Metabolism in Blood Pressure: Can B Vitamin Intervention Address the Genetic Risk of Hypertension Owing to a Common Folate Polymorphism?

**DOI:** 10.1093/cdn/nzz102

**Published:** 2019-09-16

**Authors:** Helene McNulty, J J Strain, Catherine F Hughes, Kristina Pentieva, Mary Ward

**Affiliations:** Nutrition Innovation Centre for Food and Health, School of Biomedical Sciences, Ulster University, Coleraine, Northern Ireland, United Kingdom

**Keywords:** blood pressure, hypertension, hypertension in pregnancy, one-carbon metabolism, MTHFR, folate, riboflavin, single nucleotide polymorphism, gene–nutrient interaction, personalized nutrition

## Abstract

Hypertension in adulthood is recognized as the leading risk factor contributing to mortality worldwide, primarily from cardiovascular disease, whereas hypertension in pregnancy leads to serious adverse fetal and maternal outcomes. This article explores the under-recognized role of one-carbon metabolism in blood pressure (BP) and the potential for folate-related B vitamins to protect against hypertension. Genome-wide association studies and clinical studies provide evidence linking the 677C→T polymorphism in the gene encoding methylenetetrahydrofolate reductase (MTHFR) with BP and increased risk of hypertension and hypertension in pregnancy. A novel role for riboflavin (the MTHFR cofactor) has recently emerged, however, with evidence from randomized trials that supplemental riboflavin can lower BP specifically in adults with the variant *MTHFR* 677TT genotype. Further studies are required to elucidate the biological mechanisms linking one-carbon metabolism with BP and explore the effect of riboflavin in modulating the genetic risk of hypertension in early and later life.

## Introduction

Hypertension is typically defined as a systolic/diastolic blood pressure (BP) of ≥140/90 mm Hg. It is the leading risk factor contributing to mortality worldwide, primarily from cardiovascular disease (CVD). Hypertension in pregnancy is of concern because it can lead to serious hypertensive disorders with major adverse consequences for fetal and maternal health. The development of hypertension through the lifecycle is linked with a number of well-recognized nutrition and lifestyle factors. There is now considerable evidence from genetic and clinical studies pointing to the role of one-carbon metabolism in BP, albeit this is largely overlooked in treatment of, or prevention strategies for, hypertension. This article reviews the under-recognized role of one-carbon metabolism and the potential for related B vitamins to exert a beneficial effect in maintaining healthier BP, both in patients and in subpopulations genetically at risk of developing hypertension owing to a common folate polymorphism leading to impaired one-carbon metabolism. The global health impacts of a novel gene–nutrient interaction in preventing and treating hypertension and hypertension in pregnancy will be considered, along with identification of future research priorities in this area.

## Global Burden of Hypertension and Hypertension in Pregnancy

Hypertension in adulthood is a global public health issue, estimated to affect >1 billion people (1), and is the leading risk factor contributing to mortality worldwide, primarily from CVD, particularly stroke (1–3). Each 20-mm Hg rise in systolic BP (or 10-mm Hg rise in diastolic BP) is associated with a doubling in the risk of CVD (4). The economic impacts for countries worldwide are

substantial. In the United States alone, the direct and indirect health care costs associated with hypertension are estimated at $48.6 billion and predicted to increase to $274 billion by 2030 (5).

Hypertension affects 10–15% of pregnancies and can lead to serious hypertensive disorders of pregnancy, which are recognized as the major causes of fetal and maternal morbidity and mortality worldwide (6, 7). In a notable systematic review with meta-analysis using data from 55 studies in 25 different countries, chronic hypertension (i.e., pregestational hypertension of any cause) was associated with significantly higher risks of pre-eclampsia and all other pregnancy complications, cesarean delivery, and perinatal death (8). Women with chronic hypertension in US studies were estimated to have an ∼3-fold increased risk of preterm delivery (i.e., before 37 weeks of gestation), birth weight <2500 g, and neonatal intensive care admission, and a 4-fold increased risk of perinatal death compared with the US general pregnancy population (8). Pre-eclampsia (hypertension combined with proteinuria developing in later pregnancy) is one of the most severe hypertensive disorders of pregnancy, posing a high risk of life-threatening outcomes for both mother and child (9).

Apart from the immediate adverse consequences of hypertension during pregnancy, also of concern are the long-term impacts on the mother's health. One report estimated a 65% increased risk of early adult all-cause and cause-specific mortality in women with a history of hypertension in pregnancy compared with women normotensive during pregnancy (10). There may also be implications of hypertension in pregnancy in relation to the longer-term health of the offspring. The ALSPAC (Avon Longitudinal Study of Parents and Children) reported that systolic and diastolic BP were higher in adolescent offspring (*n* = 4438) of mothers with gestational hypertension compared with mothers without hypertensive disorders of pregnancy, after adjustment for potential confounders (11).

## Preventing and Treating Hypertension

Given the significant consequences of untreated hypertension on health throughout life, effective management of hypertension should be a public health priority (12). A number of potentially modifiable factors are recognized to contribute to the development and progression of hypertension, including poor diet, obesity, smoking, alcohol, and physical inactivity (13). Thus, obvious targets for public health strategies to prevent hypertension involve interventions aimed at promoting weight reduction, increased physical activity, decreased alcohol consumption, dietary sodium restriction, or whole dietary approaches such as the Dietary Approaches to Stop Hypertension (DASH) diet (14, 15). In hypertensive patients, BP is much more effectively lowered by antihypertensive medications (typically prescribed in combinations), including calcium channel blockers, thiazide diuretics, angiotensin-converting enzyme inhibitors, and β-blockers.

There are clear benefits of treating and preventing hypertension. Reducing BP is proven to decrease CVD risk and mortality (1, 5), with estimates that lowering systolic BP by as little as 2 mm Hg can decrease CVD risk by 10% (4). In addition, major recent reports from the United States and Europe have highlighted the importance of targeting lifestyle and treatment strategies at the individual level in order to improve cardiovascular health (5, 12). In this regard, genome-wide association studies (GWASs) emerging in the past decade have enabled genes linked with BP to be identified (16, 17), but as yet no drug therapies have emerged to offer personalized treatment of hypertension based on targeting relevant genetic risk factors. Treating hypertensive conditions of pregnancy can be particularly challenging because certain antihypertensive medications are not recommended in pregnancy, with evidence that several are linked with an increased risk of a range of neonatal complications, including intrauterine growth restriction, fetal bradycardia and distress, neonatal hypoglycemia, and congenital malformations (18). Therefore, the identification of effective nondrug approaches for managing hypertension in pregnancy could be particularly beneficial.

## Evidence Linking One-Carbon Metabolism with Hypertension-Related Health Outcomes

### Functional roles of B vitamins within one-carbon metabolism

Folate is required for one-carbon metabolism, a network of pathways involved in the transfer and utilization of one-carbon units required for DNA and RNA biosynthesis, amino acid metabolism, and methylation processes. Reduced folates in various cofactor forms interact closely with vitamin B-12, vitamin B-6, and riboflavin within this network (19). Upon entering the one-carbon cycle, tetrahydrofolate (THF) acquires a carbon unit from serine in a vitamin B-6–dependent reaction to form 5,10 methyleneTHF, a folate cofactor that once generated has various fates. It is either converted to 5-methyltetrahydrofolate (5-methylTHF), or serves as the one-carbon donor in the synthesis of nucleic acids, where it is required by thymidylate synthetase in the conversion of deoyxuridine to deoxythymidine for pyrimidine biosynthesis or is converted to other folate cofactor forms required for purine biosynthesis.

Methylenetetrahydrofolate reductase (MTHFR) is the riboflavin-dependent enzyme that catalyzes the reduction of 5,10 methyleneTHF to 5-methylTHF. The latter folate form is required by methionine synthase for the vitamin B-12–dependent conversion of homocysteine to methionine with the generation of unsubstituted THF. Once formed, methionine is activated by ATP to generate S-adenosylmethionine, typically referred to as “the universal methyl donor,” because it is required for numerous methylation reactions by donating its methyl group to >100 methyltransferases involved in the regulation of essential biological processes, including the methylation of DNA, proteins, neurotransmitters, and membrane phospholipids (19). To summarize, effective folate functioning within the one-carbon cycle requires essential interaction of folate with vitamin B-12, vitamin B-6, and riboflavin. Suboptimal status of any of these B vitamins, or polymorphisms in related genes, can lead to impaired one-carbon metabolism, even if dietary folate intakes are adequate.

### One-carbon metabolism and adverse pregnancy outcomes

Folate is particularly important in pregnancy and fetal development owing to its essential role in cell division and tissue growth. The role of maternal folate in early pregnancy in preventing neural tube defects is well recognized, but it has a number of other roles throughout pregnancy with important impacts for maternal and offspring health. Historical reports from the early 1930s documenting the discovery of human folate deficiency first highlighted the importance of folate at this stage of the lifecycle and described a fatal anemia in pregnant women in India; this was subsequently proven to be responsive to treatment with food sources of the vitamin (20). It is now known that clinical deficiency of folate causes megaloblastic anemia (characterized by immature, enlarged blood cells reflecting impaired DNA synthesis), a condition that can be reversed with folic acid treatment (21). It is estimated that up to 24% of pregnancies in Asia, Africa, and South America are affected by folate-related anemia in the absence of maternal supplementation with folic acid (22), whereas supplementation prevents the decline in maternal folate concentrations that typically occurs throughout pregnancy (23, 24) and thus prevents the occurrence of megaloblastic anemia of pregnancy (25, 26). Because folate is required for the remethylation of homocysteine to methionine, plasma homocysteine concentrations are invariably found to be elevated with deficient or low folate status and are effectively lowered in response to intervention with folic acid alone or in combination with related B vitamins (27).

There is considerable evidence to link low folate status (and/or elevated homocysteine concentrations) with an increased risk of adverse pregnancy outcomes including gestational hypertension (28), pre-eclampsia (29–31), placental abruption (32), pregnancy loss (33, 34), low birth weight, and intrauterine growth restriction (35). Some (28, 36, 37), but not all (38), studies suggest benefits of folic acid supplementation in preventing gestational hypertension and pre-eclampsia. Of note, a recent report provided evidence from a randomized multicenter trial that supplementation with high-dose folic acid beyond the first trimester has no beneficial effect on pre-eclampsia in women at high risk of this condition (39). Inconsistencies in the evidence may however be explained, at least to some extent, by genetic differences between populations. As discussed below, there is emerging evidence from this center and elsewhere showing that the common C677T polymorphism in the gene encoding the folate-metabolizing enzyme MTHFR is implicated in the development of hypertension and hypertension in pregnancy (40, 41).

### One-carbon metabolism and CVDs

Considerable evidence, accumulated over many years, links one-carbon metabolism with CVD, especially stroke (42). Most relevant studies in this area have focused on plasma homocysteine as the relevant risk factor leading to CVD, but it is likely that folate and metabolically related B vitamins have protective roles in CVD that are independent of their homocysteine-lowering effects. Population data (43) and randomized trials (44) provide strong evidence that intervention with folic acid can significantly reduce stroke risk, particularly in people with no previous history of stroke (45).

Since it was first described in 1995 (46), the common C677T polymorphism in *MTHFR* has been linked with several adverse health outcomes, including an increased risk of CVD by up to 40% (40). There is, however, a large geographical variation in the extent of excess CVD risk associated with this folate polymorphism (47–49), pointing to the involvement of a gene–environment interaction, and the evidence generally shows stronger associations for studies investigating the risk of stroke than for those on heart disease (40, 50, 51).

### One-carbon metabolism, MTHFR, and BP

Folate and related B-vitamins, through functioning as one-carbon donors, may be protective against CVD through mechanisms that are not necessarily related to homocysteine. In particular, as discussed below, emerging evidence suggests that riboflavin, the MTHFR cofactor, plays an important role in modulating BP.

A link between the *MTHFR* C677T polymorphism and BP was first reported >20 y ago in a small study which found higher mean systolic/diastolic BP in middle-aged Japanese men with the variant TT genotype (147/91 mm Hg) than in those with the CT (134/81 mm Hg) or CC (133/79 mm Hg) genotypes (52). This evidence was largely overlooked for many years, perhaps because homocysteine was the phenotype of interest. More recently, GWASs and clinical studies have provided separate lines of evidence to link one-carbon metabolism—and specifically MTHFR—with BP. In 1 GWAS, 2.5 million single nucleotide polymorphisms were tested and 8 genetic loci identified as being associated with BP, including a region near the *MTHFR* gene (16), a finding confirmed in subsequent GWASs (53, 54). As reviewed elsewhere (40), the available evidence from meta-analyses of clinical studies indicates that the *MTHFR* 677C→T polymorphism is associated with an increased risk of hypertension and hypertension in pregnancy by up to 87% (41, 55, 56), with reported ORs from meta-analyses ranging from 1.36 (95% CI: 1.20, 1.53) to 1.87 (95% CI: 1.31, 2.68), for worldwide and Chinese populations, respectively (41, 57, 58).

The variant *MTHFR* 677TT genotype has a reported frequency of 10% worldwide, but this shows marked geographical variation, ranging from 4–18% in the United States, 4–26% in European populations (increasing north to south), and 20% in northern China to as high as 32% in Mexico, whereas the lowest TT genotype frequencies are found in populations of African ancestry (59). There is also marked ethnic variation within countries; thus, in certain ethnic groups within China and Mexico, even higher TT genotype frequencies are reported (60, 61), which are, in turn, associated with higher rates of hypertension (62).

## Relevant Nutrient–Nutrient and Gene–Nutrient Interactions within One-Carbon Metabolism

### Riboflavin

Riboflavin has 2 cofactor forms, FMN and FAD, which are essential for numerous oxidation-reduction reactions and thus required in the metabolism of energy, certain drugs, and toxins and in supporting cellular antioxidant potential (63). Of note, riboflavin-dependent metabolism involves interaction with a number of other nutrients including iron (64). Of particular relevance to one-carbon metabolism is the close metabolic interaction of riboflavin with vitamin B-6 (65), where riboflavin is required (as FMN) for the generation in tissues of the active vitamin B-6 coenzyme form pyridoxal 5’ phosphate (PLP) from pyridoxine phosphate by pyridoxine-phosphate oxidase (PPO). In animals, PPO activity was shown to be responsive to changes in riboflavin intake and lower PLP concentrations were found with riboflavin deficiency (65). In humans, the metabolic dependency of vitamin B-6 on riboflavin status was demonstrated in a study from our center showing that riboflavin supplementation of older adults not only improved the biomarker status of riboflavin, but also led to increased PLP concentrations (66).

Also within one-carbon metabolism, riboflavin plays an important role in folate recycling where it acts in the form of FAD as a cofactor for MTHFR in the conversion of 5,10 methyleneTHF to 5-methylTHF. The importance of riboflavin within the one-carbon metabolic network is perhaps most evident in individuals with the variant 677TT genotype in *MTHFR*, resulting in a thermolabile enzyme with reduced activity (46). Molecular studies demonstrated that the loss of MTHFR activity that occurs with the TT genotype is the result of an increased propensity for the variant enzyme to dissociate from its FAD cofactor (67, 68). In humans, the typical phenotype in adults with the variant TT genotype is one of elevated plasma homocysteine (46), along with low folate concentrations (69), and studies show that the homocysteine phenotype is most pronounced when the TT genotype occurs in combination with low folate (70) or riboflavin (71, 72) status. Marked lowering of plasma homocysteine however occurs in response to riboflavin supplementation specifically in adults with the TT genotype, an effect not found in those with the CC or CT genotypes (73). The genotype-specific responsiveness of homocysteine to riboflavin intervention may indicate that optimizing riboflavin status can stabilize the variant enzyme and thus restore MTHFR activity in vivo. Of greater relevance to public health, however, is emerging evidence that riboflavin interacts with MTHFR to influence BP and hypertension risk.

### Novel genotype-specific role of riboflavin in BP

An entirely novel role of riboflavin as an important modulator of BP has emerged in recent years, specifically in genetically at risk individuals owing to the C677T polymorphism in *MTHFR* (40). Studies from our center show significantly higher systolic and diastolic BP in adults homozygous for this polymorphism but this phenotype appears to be highly responsive to riboflavin intervention (74–76).

In the first of 3 randomized trials, premature CVD patients (mean age of 53 y) with the variant *MTHFR* 677TT genotype were found before intervention to have significantly higher systolic/diastolic BP (143/86 mm Hg) than age- and sex-matched patients with the CC (131/80 mm Hg) or CT (133/83 mm Hg) genotypes (74). In response to supplementation with riboflavin (1.6 mg/d for 16 wk), however, BP decreased by a mean of 14 mm Hg (systolic BP) specifically in patients with the TT genotype, with no BP response in the CC or CT genotype groups (74). When this cohort of premature CVD patients was followed up 4 y later, those with the TT genotype were hypertensive at baseline, despite marked changes in the number and type of antihypertensive drugs being prescribed since the initial investigation (following changes in clinical guidelines for hypertension), and goal BP (i.e., systolic/diastolic BP ≤140/90 mm Hg) was achieved only in response to riboflavin intervention (75). In a third trial, hypertensive adults without overt CVD were investigated (76). Despite being prescribed multiple classes of antihypertensive drugs, >60% of participants with the variant TT genotype in *MTHFR* were found to be hypertensive at baseline, but after riboflavin intervention for 16 wk (during which time antihypertensive drug treatment remained unchanged), BP significantly decreased and there was a marked improvement in BP control (76). Together these trials show that targeted riboflavin supplementation of genetically at-risk adults can effectively lower BP and improve BP control in hypertensive patients, with or without overt CVD, independently of concurrent antihypertensive drug use.

### Do other relevant B vitamins have a role in BP?

Red blood cell folate (RBC) concentrations are found to be significantly lower in people with the *MTHFR* 677TT genotype, perhaps indicating a higher requirement for folate in order to normalize folate metabolism in these individuals (69). It is possible that in people with the TT genotype, supplementation with the folate derivative 5-methylTHF may achieve a better folate biomarker response than that obtained with an equivalent dose of folic acid (the synthetic vitamin form), because 5-methylTHF would bypass the relevant MTHFR-dependent step in folate metabolism, but there is no direct evidence for such an effect in human studies. Moreover, given the dependency of MTHFR on not only folate, but also riboflavin, the combined effect of these vitamins may have a greater effect on the biomarker response than intervention with folate alone. It is also possible that any corresponding effect on the BP phenotype arising from correcting MTHFR activity in TT individuals (among whom normal folate recycling is impaired) that occurs with riboflavin only could be further enhanced by combining riboflavin with 5-methylTHF. This remains entirely speculative, however, because no human study to date has investigated the genotype-specific effect on BP of riboflavin and 5-methylTHF in combination.

Apart from any genotype-specific BP-lowering effects, it is unlikely that supplementation with folate-related B vitamins in adults generally is beneficial in maintaining healthy BP. Numerous randomized trials involving intervention with folic acid (typically in combination with vitamin B-12 and vitamin B-6) aimed at lowering homocysteine concentrations and CVD showed little or no corresponding BP response (77, 78).

### Mechanisms explaining the role of one-carbon metabolism in BP

The biological mechanisms linking one-carbon metabolism with BP and explaining the modulating role of riboflavin are unclear. In individuals with the *MTHFR* 677TT genotype, MTHFR enzyme activity appears to be particularly sensitive to changes in riboflavin status (71–73). It could be speculated that in TT-genotype individuals there is a higher capacity to replace inactivated enzyme with optimal riboflavin than with low riboflavin status, or that higher riboflavin status prevents the FAD cofactor from leaving the active site or allows its quick replacement, thus stabilizing the variant form of the enzyme. Riboflavin requirements may therefore be higher in individuals with the TT genotype in order to sustain normal MTHFR activity (71, 73), although this remains to be specifically demonstrated.

As reviewed elsewhere (40), the MTHFR–BP relation is likely to involve the potent vasodilator nitric oxide (NO) via an effect on endothelial function. Vascular tissue concentrations of 5-methylTHF (the product of the MTHFR reaction) were associated with NO bioavailability and improved endothelial function in patients undergoing coronary artery bypass graft surgery, and were found to be lower in those patients with the *MTHFR* 677TT genotype (79, 80). Stabilizing the variant MTHFR enzyme in vivo with supplemental riboflavin (73) could at least partially restore 5-methylTHF concentrations in vascular cells in the TT genotype. This in turn would improve NO availability and endothelial function, and thus lower BP specifically in individuals with the TT genotype. Restoration of cellular concentrations of 5-methylTHF with riboflavin supplementation may also correct any imbalance of THF derivatives owing to this polymorphism, and specifically the accumulation of formylTHF relative to 5-methylTHF that is reported in RBCs in individuals with the TT genotype (81). The impaired folate metabolism that occurs in this polymorphism may also affect the activity of dihydrofolate reductase which, along with dihydrobiopterin reductase, contributes to the generation of tetrahydrobiopterin from dihydrobiopterin (82). Tetrahydrobiopterin is an essential cofactor in the coupling of reduction and oxidation reactions catalyzed by nitric oxide synthase (eNOS). When coupled with tetrahydrobiopterin, eNOS produces NO from arginine and NAD(P)H, inducing vasodilation, but with accumulation of dihydrobiopterin, eNOS activity leads to the generation of superoxide (83, 84). In summary, by correcting MTHFR activity with riboflavin supplementation in the TT genotype, endothelial function could be improved through increased bioavailability of NO, via increased vascular concentrations of 5-methylTHF and/or tetrahydrobiopterin.

Whatever the mechanism explaining the role of one-carbon metabolism in BP, it is unlikely to involve homocysteine. Although significant associations of plasma homocysteine with BP have been reported in several observational studies, no BP response was found in trials designed to lower homocysteine as a means to reduce CVD (77, 78). Mechanistic studies are required to elucidate the biological perturbation that leads to higher BP with the common *MTHFR* C677T polymorphism and to understand how riboflavin can rescue this phenotype and thus potentially protect against the development of hypertension.

## Health Impacts, Challenges, and Research Priorities

### Health impacts of a novel gene–nutrient interaction in BP

Hypertension in adulthood continues to be the major risk factor for cardiovascular death in every region of the world (85). Effective lowering of BP is, however, proven to be highly beneficial in reducing cardiovascular mortality (5, 86, 87). In 2017, the American Heart Association published new guidelines for the prevention, detection, and management of elevated BP in US adults and lowered the threshold defining hypertension, from the existing BP level (systolic/diastolic) of >140/90 mm Hg to >130/80 mm Hg. These changes were introduced primarily as a result of compelling findings from the SPRINT (Systolic Blood Pressure Intervention Trial) study, a large multicenter study that reported lower rates of fatal and nonfatal major cardiovascular events and death from any cause, in response to intensive compared with standard BP-lowering treatment (88). This evidence has led to calls for newer approaches, including novel combination therapies and nonpharmacological solutions (89).

Hypertensive disorders of pregnancy are internationally categorized into 4 clinical categories: gestational hypertension (pregnancy-induced hypertension after 20 weeks of gestation); chronic hypertension (pregestational hypertension of any cause); pre-eclampsia/eclampsia; and chronic hypertension with superimposed pre-eclampsia. The combined impact of these disorders is estimated to account for 14% of maternal deaths worldwide (9). Pre-eclampsia in particular remains a leading cause of maternal and perinatal mortality and morbidity globally, particularly in low- and middle-income countries (7). In combination with a rising maternal age in many countries, hypertension in pregnancy puts the lives of women and their infants at risk and imposes an increasing health care burden on society. There have been recent calls therefore for effective, safe, and affordable treatments and prevention strategies (90).

Given the considerable burdens of hypertension and hypertension in pregnancy and the proven benefits of effective treatment, important health impacts throughout the lifecycle for relevant subpopulations globally could arise from implementing interventions based on the evidence reviewed here, showing a novel and genotype-specific role for riboflavin in lowering BP. The magnitude of BP lowering demonstrated in the variant *MTHFR* genotype in response to riboflavin (by a mean decrease of 6–14 mm Hg in systolic BP across 3 separate trials) (74–76) is clinically significant, considering that each 2-mm Hg lowering of systolic BP is estimated to decrease CVD risk by 10% (4). Furthermore, the genotype-specific BP response to riboflavin occurs independently of antihypertensive drugs (74–76), and, as currently prescribed, these appear to be associated with poorer BP control in patients with the *MTHFR* 677TT genotype, whereas achievement of target BP can be greatly enhanced in these at-risk patients with supplemental riboflavin (76). Other genetic factors are implicated in the development of hypertension (16, 53, 54), but the common *MTHFR* C677T polymorphism is the only genetic factor linked with hypertension that offers a personalized management option, via optimizing riboflavin, the MTHFR cofactor.

### Optimizing riboflavin status—a global concern

Riboflavin deficiency is a significant problem in developing countries (91). Suboptimal riboflavin status on a more widespread basis may also exist across the developed world, but this is generally unrecognized because riboflavin biomarkers are rarely measured in human studies (92). The United Kingdom and Ireland are among the very few countries worldwide to have included a riboflavin biomarker as part of their population-based nutrition surveys (93, 94). In both national nutrition surveys, the biomarker status of riboflavin was measured using the erythrocyte glutathione reductase activation coefficient (EGRac) assay, widely considered to be the gold-standard measure of status (95). This coefficient is expressed as the ratio of glutathione reductase activity in lysed red cells with, to without, the *in vitro* addition of FAD and provides a measure of enzyme saturation with its riboflavin-derived cofactor. A low EGRac value is generally considered to be normal, whereas higher values indicate suboptimal status, but there is no universal agreement as to the precise EGRac cutoffs to categorize deficient and low riboflavin status.

Some concern has however been expressed regarding the high proportion of healthy adults with EGRac values indicative of suboptimal biomarker status of riboflavin, as assessed in both the British and Irish population-based surveys. On the limited available evidence, therefore, suboptimal riboflavin status may be more widespread than is generally recognized in populations globally because of the current reliance on dietary data only in nutrition surveys in most countries (including in the United States and Canada), without corresponding riboflavin biomarkers. There is a need to measure the biomarker status of riboflavin in population surveys, and to demonstrate the functional and health effects of riboflavin, covering the range from deficient to suboptimal to optimal status.

## Conclusions

The evidence was reviewed linking one-carbon metabolism and related B vitamins with BP, risk of hypertension, and hypertension in pregnancy. These are significant public health concerns for populations worldwide. Homozygosity for the C677T polymorphism in *MTHFR*, affecting 1 in 10 adults globally, is associated with higher BP, but emerging evidence shows that riboflavin (the MTHFR cofactor) exerts an important modulating effect on the BP phenotype, with riboflavin supplementation proven to lower BP in adults with the variant TT genotype. The finding that the BP phenotype associated with this common folate polymorphism is modifiable by riboflavin may have important clinical and public health impacts. For hypertensive patients or subpopulations worldwide with the variant *MTHFR* 677TT genotype, enhancing riboflavin status could offer a personalized option to treat, delay, or prevent the development of high BP. The health impacts of intervention with riboflavin can be anticipated to be greatest in those countries worldwide, including Mexico and northern China, with the highest reported frequencies of the variant genotype. Other relevant genes for BP have been identified in GWASs, but this folate polymorphism is the only genetic factor linked with hypertension that offers a personalized management option, via optimizing riboflavin. Further investigations are required to explore the underpinning biological mechanisms linking one-carbon metabolism with BP and to more fully investigate the effect of riboflavin and other relevant B vitamins in preventing hypertension and hypertension in pregnancy in adults genetically at risk owing to this polymorphism. Large-scale studies are also required to investigate the long-term health outcomes, particularly in relation to CVD and hypertensive disorders of pregnancy, of targeted B vitamin intervention at various stages of the lifecycle.

## Notes

The research described in this review was supported in part by governmental funding from the Irish Department of Agriculture, Food and the Marine and the Health Research Board (under the Food Institutional Research Measure initiative) and from the Northern Ireland Department for Employment and Learning (under its Strengthening the All-Island Research Base initiative); and by DSM Nutritional Products.

Author disclosures: CFH and KP, no conflicts of interest. HM, JJS, and MW hold an international patent on the use of riboflavin in the treatment of blood pressure.

None of the entities providing support were involved in the writing of this article.

Abbreviations used: 5-methylTHF, 5-methyltetrahydrofolate; BP, blood pressure; CVD, cardiovascular disease; EGRac, erythrocyte glutathione reductase activation coefficient; eNOS, endothelial nitric oxide synthase; GWAS, genome-wide association study; MTHFR, methylenetetrahydrofolate reductase; NO, nitric oxide; PLP, pyridoxal 5’phosphate; PPO, pyridoxine-phosphate oxidase; RBC, red blood cell; THF, tetrahydrofolate.
